# The College of Nursing (Limited by Guarantee)

**Published:** 1920-11-27

**Authors:** 


### The College of Nursing
### (Limited by Guarantee).

BIRMINGHAM THREE COUNTIES CENTRE.

On Tuesday, November 23, in the Lecture Theatre of the General Hospital, Birmingham (by kind permission of the Governors), Mr. G. P. Mills, F.R.C.S., gave an interesting lecture on "Some Surgical Lessons from the War." Mr. Mills spoke of the importance of the early treatment of septic wounds and of the value of bacteriology as a determinate factor in dealing with them; he also referred to the excellent results obtained by means of the Carrel-Dakin methods, and the changed conditions under which fractures are treated. Mr. Mills referred briefly to the tapping of fluid in the pleural cavity by means of oxygen replacement and the improved method of drawing an empyema. He concluded a most interesting and instructive lecture by impressing upon his audience the importance of observing the local signs of the onset of tetanus.

